# Enteral versus parenteral nutrition in auto-HCT: a randomized controlled trial on clinical outcomes and gut microbiome dynamics

**DOI:** 10.1007/s00520-025-09882-z

**Published:** 2025-09-19

**Authors:** Hannah R. Wardill, Lenneke F. J. van Groningen, Mohsen Dorraki, Eva B. D. Molendijk, Doris Kalter, Ana Rita Da Silva Ferreira, Alexander Kurilshikov, Feargal J. Ryan, Johan W. Verjans, Hermie J. M. Harmsen, Wim J. E. Tissing, Walter J. F. M. van der Velden, Nicole M. A. Blijlevens

**Affiliations:** 1 Department of Hematology, Radboud University Medical Center, Nijmegen, The Netherlands; 2 Supportive Oncology Research Group, Precision Cancer Medicine Theme, South Australian Health and Medical Research Institute, North Terrace, Adelaide, Australia; 3 Radboud Institute of Health Sciences, Radboud University Medical Center, Nijmegen, The Netherlands; 4 Australian Institute for Machine Learning (AIML), Adelaide, Australia; 5 South Australian Health and Medical Research Institute (SAHMRI), Adelaide, Australia; 6 Department of Medical Microbiology, University of Groningen, University Medical Center Groningen, Groningen, The Netherlands; 7 Department of Genetics, University of Groningen, University Medical Center Groningen, Groningen, The Netherlands; 8 Flinders Health and Medical Research Institute, Flinders University, Bedford Park, Australia; 9 Princess Maxima Centre for Paediatric Oncology, Utrecht, The Netherlands; 10 School of Biomedicine, Faculty of Health and Medical Sciences, The University of Adelaide, Adelaide, Australia; 11 Department of Paediatrics, University of Groningen, University Medical Centre Groningen, Groningen, The Netherlands

**Keywords:** Haematopoietic cell transplantation, HCT, Nutrition, Apoptosis, Gastrointestinal mucositis, Citrulline, Gut microbiome

## Abstract

Disruption of the gut microbiome is a common consequence of chemotherapy, linked with detrimental treatment outcomes (e.g. sepsis), especially in haematopoietic stem cell transplant (HCT) recipients. Preclinical data suggest that enteral nutrition (EN) is superior to parenteral nutrition (TPN), minimising gut atrophy and promoting eubiosis; yet, TPN continues to be used. Here, we evaluated the clinical effects of EN and TPN in autologous HCT recipients and their influence on gut microbiome dynamics. Despite efforts to optimise delivery, EN was poorly tolerated and ultimately proved unfeasible. As such, we turned our attention to analysing microbial dynamics in our study cohort and confirmed preclinical reports that epithelial apoptosis drives gut microbiome disruption. Machine learning models predicted microbial composition by tracking plasma citrulline trajectories, a biomarker of enterocyte mass. These findings suggest that (i) monitoring citrulline may be able to identify patients at risk of potentially lethal HCT complications associated with gut microbiome disruption, and that (ii) preserving epithelial integrity could support microbial resilience by minimising the production of caspase-dependent metabolites. Dutch Trial Register: NL4069. Data registered: 19–11-2013. www.trialregister.nl

**Supplementary Information:**

The online version contains supplementary material available at 10.1007/s00520-025-09882-z.

## Introduction

Autologous haematopoietic stem cell transplantation (auto-HCT) is a curative approach used to treat a range of malignant blood cancers, including multiple myeloma and lymphoma, in which patients are conditioned with chemotherapy prior to reconstitution with haematopoietic stem cells. Due to the intensity of conditioning treatment, which usually involves high-dose chemotherapy, auto-HCT recipients develop a range of acute and chronic complications including oral mucositis, nausea/vomiting, infection and diarrhoea. Malnutrition is one of the most challenging complications in auto-HCT, associated with inferior clinical outcomes including increased risk of infection, service provision and non-relapse mortality as well as decreased overall survival [1–4]. International guidelines differ in recommendations on how to best assess, prevent and treat malnutrition, especially in auto-HCT recipients with severe gastrointestinal mucositis (GI-M), which compromises mucosal surface area and function. Most guidelines recommend the provision of clinical nutrition, either through enteral or parenteral routes. However, a Cochrane collaboration review evaluating enteral vs. total parenteral nutrition (EN vs. TPN) concluded that insufficient data exist to make a proper comparison between these feeding routes in managing malnutrition in auto-HCT [5]. As a result, enormous variability exists in clinical practice as evidenced by a recent survey conducted on behalf of the Nurses Group and Transplant Complications Working Party of the European Society for Blood and Marrow Transplantation. In this cross-section survey of *N* = 108 nurses across 108 European transplant centres in 16 countries, highly variable practices were reported between countries and centres, with low adherence to available guidelines [6]. Generally, however, TPN was most commonly reported across the respondents (81.5%), with EN (tube feeding) use reported in 28.7% of respondents. Although this survey did not stratify results based on auto- vs. allogeneic-HCT (allo-HCT) practices, the results mirror findings from other studies in allo-HCT recipients where TPN is more feasible [7] and preferred by medical teams [7], reflecting inherent, yet unsubstantiated concerns regarding excessive gastrointestinal complications associated with EN [8] and patient preference [9].

Despite the historical and ongoing preference for TPN, preclinical studies suggest a beneficial role of EN in auto-HCT patients with G-IM [10, 11], with EN preserving mucosal integrity and promoting gut microbiome structure [12–19]. This trial was therefore designed to evaluate the effects of EN (via nasogastric tube, NGT) vs. TPN on nutritional status in auto-HCT recipients and the development of transplant-related complications, including changes in the gut microbiota. We hypothesise that EN will improve clinical outcomes and promote microbial stability compared to TPN. Given the highly protocolised nature of this trial (including restricted antibiotic use), repeated biospecimens were also collected to explore the interplay between G-IM and gut microbiome compositions, with the goal of better understanding the drivers of gut microbial disruption.

## Materials and methods

### Study design and participants

From September 2014 to January 2018, all patients ≥ 18 years subsequently referred to the Radboud University Medical Center Nijmegen in the Netherlands for auto-HCT with either high-dose melphalan (HDM) or carmustine, etoposide, cytarabine and melphalan (BEAM) conditioning were screened for eligibility. Exclusion criteria for participation in the study were severe malnutrition defined as a BMI < 18 kg/m2 and/or a serum albumin < 20 g/L, renal insufficiency defined as a creatinine level > 150 µmol/L or a creatinine clearance < 50 mL/min, use of pre- and probiotics, and pre-existing bowel diseases, e.g. short bowel syndrome, Crohn’s disease or celiac disease. Patients were randomly assigned to TPN or EN, with a 1:1 ratio, with randomisation performed independently by the Trial Data Centre of the Department of Haematology. Stratification was based on the type of conditioning (HDM or BEAM). This study was approved by the local ethics Committee (CMO region Arnhem-Nijmegen). All patients participating in the study provided written informed consent, and all procedures were conducted in accordance with the Declaration of Helsinki. This trial was registered at www.trialregister.nl as NL4069.

### Procedures of nutritional intervention

At the time of completing the study at Radboud University Medical Center, TPN was the standard of care. As such, all participating patients (both EN and TPN groups) received a central venous catheter (CVC), which was placed on the day of admission as standard clinical practice. EN was started immediately after placing the NGT on day + 4 and administered continuously in increasing amounts by an EN feeding pump. EN was started at 500 mL/24 h and increased each day by 500 mL to a maximum of 1500 or 2000 mL/24 h referred to the calculated dietary need. All patients received pantoprazole 40 mg once daily and were encouraged to continue oral intake if tolerated even whilst TPN or EN was delivered. Nutritional support (either TPN or EN) was discontinued when the total oral intake was sufficient as determined by the dietician. For further details on EN and TPN provision, please see Supplementary Methods.

### Supportive care

As standard of care during hospitalisation of auto-HCT patients, faecal samples were collected twice a week to detect colonisation with (resistant) micro-organisms, yeasts or fungi. Antibiotic use was very limited and highly protocolised to minimise inter-individual heterogeneity and its effects on the gut microbiome. Patients received ciprofloxacin 500 mg BID for antibacterial Gram-negative prophylaxis, valaciclovir 500 mg BID for herpes viruses and co-trimoxazole 480 mg QD (after engraftment) for *Pneumocystis jirovecii* prophylaxis. Fluconazole 400 mg QD was only used in those colonised with *Candida *species. Routine surveillance blood cultures were drawn from the CVC two times a week. In case of fever during neutropenia, standard additional peripheral blood and CVC cultures were taken, and empirical monotherapy with ceftazidime 2000 mg TID was started [20].

### Withdrawal of subjects

Patients were considered non-evaluable if NGT was lost within 48 h or EN was intolerable for 5 days. Since withdrawal of individual subjects due to intolerance of a NGT or EN was anticipated, study inclusion was permitted for up to a maximum of six additional patients.

### Endpoints

The primary endpoint was to determine differences in nutritional status based on change of body weight (Δbody weight) and change in mid-upper arm circumference (ΔMUAC) on day + 28 as an indicator of malnutrition (day 0 = start of conditioning). The primary outcome is presented as per intention to treat and per protocol analyses. Secondary endpoints are outlined in Supplementary Methods, with mucositis assessment in alignment with recent recommendations by the Multinational Association of Supportive Care in Cancer [21].

### Exploratory analysis of the gut microbiome

Faecal samples were collected twice weekly during hospitalisation, with the first sample taken before auto-HCT and use of prophylactic antibiotics. A final sample was collected after discharge at the outpatient clinic, ranging between days + 20 and + 90. Faecal samples (~ 6 g) were collected aseptically into Sarstedt faecal tubes and stored at − 80 °C. 16S rRNA gene sequencing was performed as per Supplementary Methods.

### Statistical analyses

Sample size calculations are based on a two-sided test using a two-way ANOVA, with a power of 80% and *α* = 0.05. We assumed a standard deviation (residual standard deviation) of 3 kg in each of the four groups (HDM + TPN, HDM + EN, BEAM + TPN, BEAM + EN). To prove a significant difference in weight loss during auto-HCT (estimated body weight reduction 5 versus 2.0 kg for TPN and EN, respectively, 28 days after start chemotherapy), 34 patients (17 in each interventional arm) were required.

Patient characteristics were compared by using Student’s* t* test. All statistical analyses (with the exception of microbiota data) were performed with SPSS for Windows (Version 25.0, 2017, IBM Corp., Chicago, IL. USA) and SAS software (Version 9.4, 2013). Categorical variables were assessed using Pearson’s chi-square test or Fisher’s exact tests. Continuous variables were assessed using non-parametric independent samples tests (Mann–Whitney *U* test). Pearson correlation coefficients were used for all correlations. Where repeated measures are used (e.g. longitudinal data), mixed models were used to determine statistically significant differences. All tests were two-tailed and *P* < 0.05 was considered statistically significant. GraphPad Prism (v10) was used to prepare figures.

In the per protocol analysis, we analysed only the patients who were ‘evaluable’. Patients were considered not evaluable (treatment failure) when there was NGT loss within 48 h after placement or when EN was not tolerated during the first 5 days. All patients were included in the intention to treat analysis, whilst only evaluable patients were included in the per protocol analysis.

## Results

### Patients

*N* = 40 patients were included in the trial, randomised 1:1 to receive EN or TPN (*N* = 20/group). *N* = 31 were evaluable (Table 1); consisting of 11 patients (35%) received EN (EN group) and 20 patients (65%) received TPN (TPN group). To provide clarity on the intention to treat (ITT) vs. per protocol (PP) cohorts, of the original *N* = 20 participants randomised to EN, *N* = 6 participants were non-evaluable. They were subsequently replaced as per our protocol permissions (*N* = 20 ITT cohort). However, a further *N* = 9 were non-evaluable due to intolerance (hence, *N* = 11 PP cohort); however, these participants could not be replaced due to ethical restrictions (Figure S1). Adherence to EN was suboptimal, with only *N* = 6 participants in the EN group receiving exclusively EN. The remaining *N* = 5 participants received TPN because of NGT loss or intolerance for EN after day 5. EN was given for a median of 11 days (range 5–22). *N* = 7 (66%) patients of the EN group used EN ≥ 10 days. No bleeding complications related to the NGT placement were observed; specifically, there were no cases of epistaxis secondary to EN. In contrast to the low uptake of EN, TPN was given to all *N* = 20 patients randomised to receive TPN, with no stopping criteria met with respect to elevated liver enzymes, hypertriglyceridemia or hyperglycemia. TPN was given for a median of 10 days (range 4–21). *N* = 13 patients (65%) of the TPN group used TPN ≥ 10 days. The rate of febrile neutropenia (and thus, ceftazidime) was comparable between groups (*N* = 10 vs. *N* = 14; EN vs. TPN, *P* = 0.18). There were no instances of oral intake during TPN; hence, there were no cases of participants consuming via TPN and oral routes concurrently.

**Table 1:** Patient demographics and clinical characteristics at baseline

Characteristic	TPN (*n* = 20)	EN (*n* = 11)
Age years—median (range)	57 (44–67)	60 (49–69)
Sex		
Male—no. (%)	15 (75)	9 (82)
Female—no. (%)	5 (25)	2 (18)
Bodyweight kg—mean ± SD	81.3 ± 13.2	86.1 ± 10.8
Length m—mean ± SD	1.76 ± 0.09	1.78 ± 0.08
BMI—mean ± SD	26.1 ± 3.6	27.3 ± 4.0
MUAC cm— mean ± SD	31.3 ± 3.2	32.2 ± 2.9
Diagnosis		
MM—no. (%)	12 (60)	6 (55)
NHL—no. (%)	8 (40)	5 (45)
Conditioning regimen—no. (%)		
HDM—no. (%)	12 (60)	6 (55)
BEAM—no. (%)	8 (40)	5 (45)
Baseline laboratory results		
Citrulline—mean ± SD	34.0 ± 10.3	36.4 ± 10.3
CRP (mg/L)—mean ± SD	3.2 ± 2.4	3.0 ± 2.4
Albumin (g/L)—mean ± SD	37.2 ± 3.1	36.6 ± 6.1
GFR (mL/min/1.73 m^2^)—mean ± SD	87.1 ± 7.0	79.9 ± 10.5
Creatinine (µmol/L)—mean ± SD	70.9 ± 11.4	83 ± 17.0
Melphalan/kg—mean ± SD	4.15 ± 1.07	4.19 ± 0.88
Calory need (kCal)—mean ± SD	2324 ± 318	2390 ± 290

*TPN* total parenteral nutrition, *EN* enteral nutrition, *BMI* body mass index, *MUAC* mid-upper-arm circumference, *MM* multiple myeloma, *NHL* non-Hodgkin lymphoma, *HDM* high-dose melphalan, *BEAM* carmustine, *CRP* C-reactive protein, *GFR* glomerular filtration rate

### Primary and secondary endpoints

**Fig. 1:**
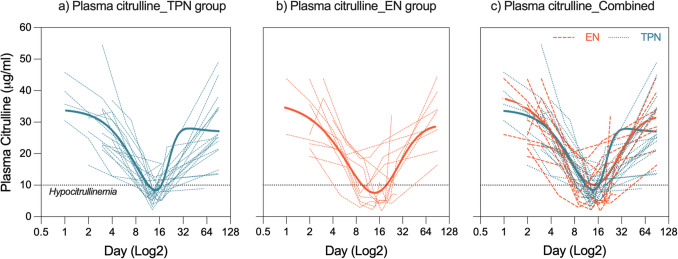
Citrulline course per individual patient TPN (**a**) and EN (**b**) groups, and combined study cohort (**c**). Solid lines show non-linear best fit curve using all data points. No statistically significant differences were observed in the per protocol analysis. Hypocitrullinemia (defined as plasma citrulline concentration of < 10 μg/mL) indicated on graphs as a horizontal-dotted line

The Δbody weight and ΔMUAC in both groups were not significantly different on day + 28. The intention to treat analysis showed that the Δbody weight on day + 28 was − 3.68 kg in the TPN group and − 4.02 kg in the EN group, *P* = 0.77. The ΔMUAC was − 1.02 cm and − 1.41 cm in the TPN and EN groups respectively, *P* = 0.43 (Table 2), nor were there any significant differences in per protocol analysis (Table 2). No significant differences were observed in plasma citrulline (GI-M biomarker, Fig. 1, Table 3), nor were there differences in any other secondary outcome measure (Table 3).

**Table 2:** Change in bodyweight and mid-upper-arm circumference

Time point	Compared with day of admission ***(intention to treat analysis)***					
	Mean weight change (kg)			Mean MUAC change (cm)		
	TPN	EN	*p value*	TPN	EN	*P value*
Day + 28	− 3.68	− 4.02	0.77	− 1.02	− 1.41	*0.43*
Day + 45	− 2.85	− 5.46	0.03	− 0.97	− 1.27	*0.56*
Day + 90	− 1.27	− 4.02	0.03	− 0.46	− 1.23	*0.13*
Time point	Compared with day of admission ***(per protocol analysis)***					
	Mean weight change (kg)			Mean MUAC change (cm)		
	TPN	EN	*p value*	TPN	EN	*P value*
Day + 28	− 3.68	− 3.96	0.77	− 1.02	− 1.63	*0.16*
Day + 45	− 2.85	− 4.29	0.17	− 0.97	− 1.32	*0.44*
Day + 90	− 1.27	− 2.89	0.12	− 0.46	− 1.36	*0.05*

*TPN* total parenteral nutrition, *EN* enteral nutrition, *MUAC* mid-upper-arm circumference

*P* < 0.05 = significant

**Table 3:** Secondary endpoints (per protocol analysis)

Secondary endpoints	TPN group (*n* = 20)	EN group (*n* = 11)	*P value*
Citrulline (µg/mL) Day + 28 Day + 45 Day + 90	21.4 ± 7.2 22.1 ± 6.3 24.7 ± 13.2	17.3 ± 9.03 24.7 ± 10.3 29.0 ± 9.99	*0.18* *0.41* *0.70*
CRP (mg/L) Day + 28 Day + 45 Day + 90	19.3 ± 57 4.3 ± 4.8 5.2 ± 9.6	17.6 ± 19.6 6.3 ± 6.2 4.6 ± 4.7	*0.92* *0.34* *0.82*
Oral mucositis Grade I (*n*, %) Grade II (*n*, %)	17 (85%) 3 (15%)	7 (63.6%) 4 (36.4%)	*0.17*
BSI (*n*, %)	6 (30%)	3 (27%)	*0.67*
Febrile neutropenia (*n*, %)	14 (70%)	10 (90%)	*0.18*
Haematological recovery (mean ± SD) (days)	17 ± 6	17 ± 5	*0.87*
Hospital stay (mean ± SD) (days)	24 ± 9	22 ± 5	*0.36*

*TPN* total parental nutrition, *EN* enteral nutrition, *BSI* blood stream infection, *SD* standard deviation

### Exploratory analysis of the gut microbiota

#### Gut microbiome changes in study cohort

**Fig. 2:**
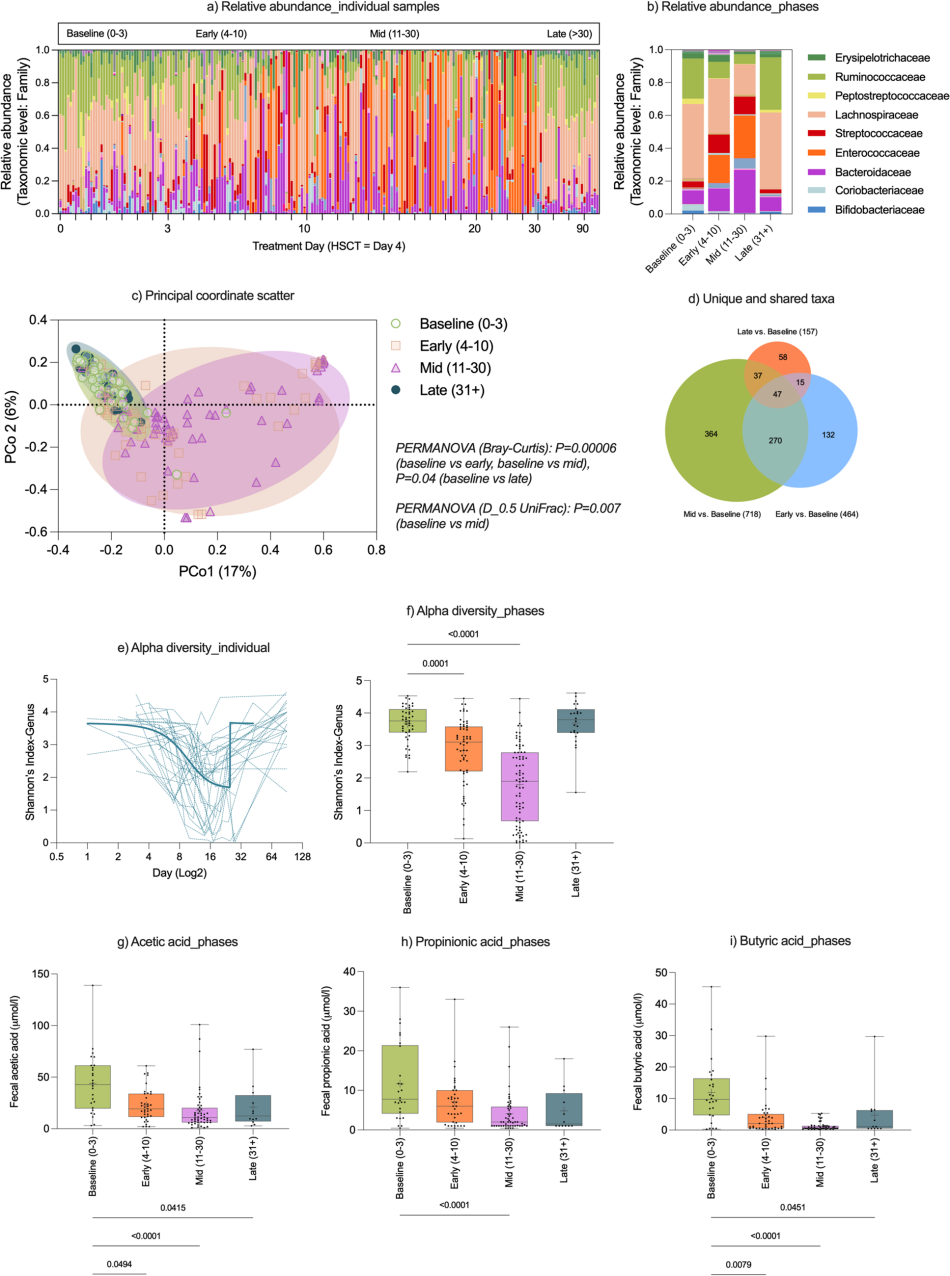
Gut microbiome composition in entire study cohort, independent of randomisation group. Gut microbiome composition (**a**, **b**) is represented as the relative abundance of bacterial taxa at the family level, shown for individual participant samples collected longitudinally during the study period (**a**) or group averages shown at distinct treatment phases (**b**). Relative abundance over time shows substantial expansion in enteric pathogens (**a**, **b**). Beta diversity (Bray–Curtis) showed a compositional shift in early, mid and late timepoints (**c**), representing unique and shared microbial taxa across treatment phases (**d**). Alpha diversity (Shannon’s index) was profoundly decreased after auto-HCT (**e**), decreasing in the early- and mid-treatment phases (**f**). This corresponded with a decrease in faecal acetic acid (**g**), propionic acid (**h**) and butyric acid (**i**)

A total of 249 faecal samples were collected from trial participants (4–9 stool samples (7 ± 1.4) per participant) from day 0 (start of conditioning therapy) to day + 90. The taxonomic composition of the gut microbiome changed dramatically after conditioning chemotherapy (Fig. 2a, b), with PERMANOVA (Bray–Curtis) confirming significant taxonomic shifts from baseline (*P* < 0.0001, relative to early- (days 0–3) and mid- (days 4–10) treatment phases; *P* = 0.04 relative to late phase, Fig. 2c). PERMANOVA (generalised UniFrac, weight = 0.5, D_0.5_UniFrac) showed a significant difference between baseline and the mid-phase (*P* = 0.007). There were 317 OTUs shared across all treatment phases, with 364 unique OTUs in the mid-phase determined using a generalised linear model differential abundance test (Fig. 2d). Differential abundance analysis of data aggregated by treatment phase identified sixty OTUs that differed significantly across phases relative to baseline (Table S2). This was predominantly in the early phase, with significant increases in OTUs with pathogenic potential from the genera *Enterococcus*, *Staphylococcus* and S*treptococcus.* Taxonomic shifts were accompanied by a decrease in alpha diversity (Fig. 2e, f), which was significantly decreased (relative to baseline) at the early (*P* < 0.0001) and mid (*P* < 0.0001) phases, as well as a decrease in faecal SCFA concentrations (Fig. 2g–i).

**Fig. 3:**
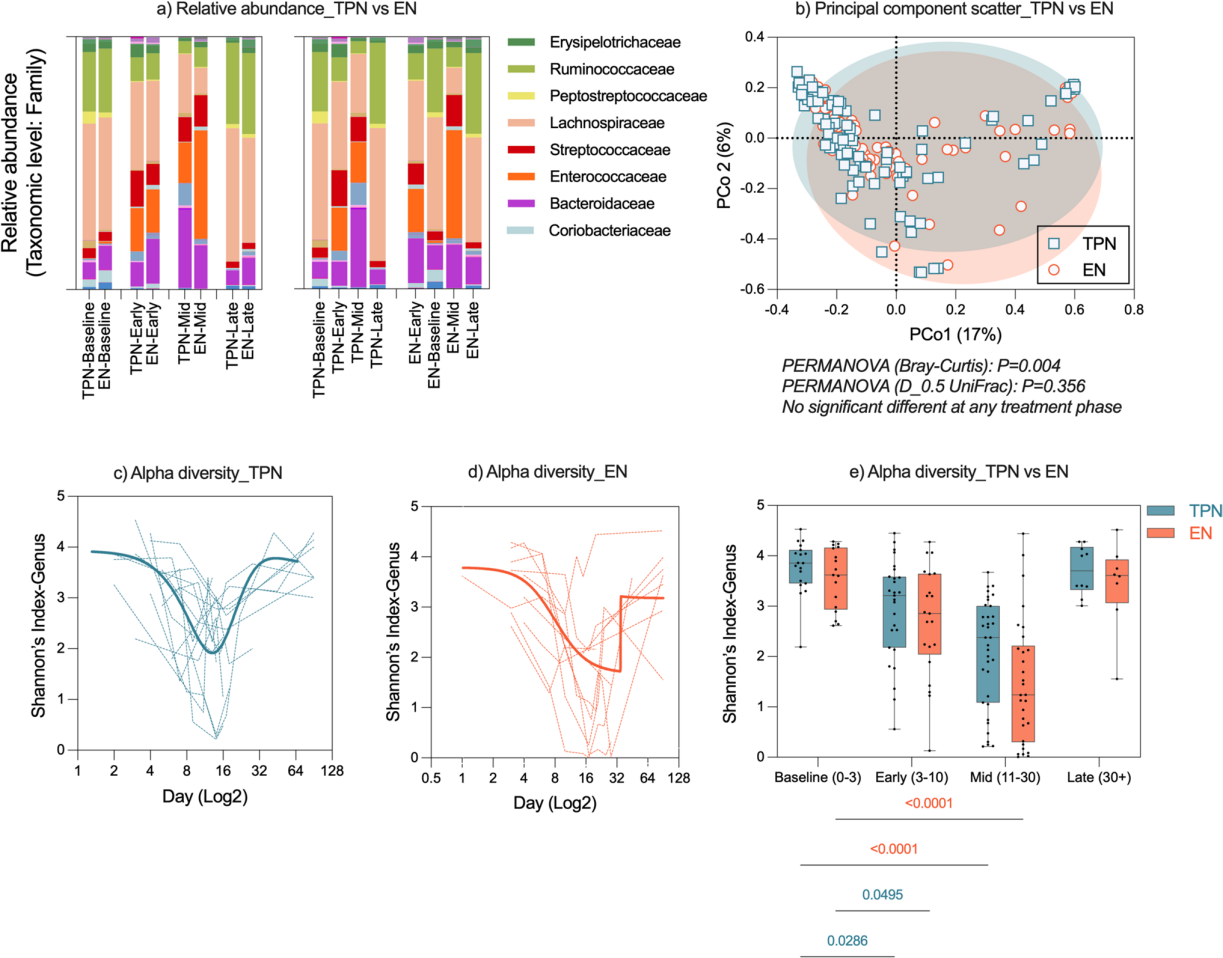
Gut microbiome composition in TPN versus EN groups. Relative abundance, shown as stacked column graphs based on the taxonomic distribution of certain bacterial taxa, in treatment phases (**a**) demonstrates unique compositional changes in the gut microbiome in TPN vs. EN participants, aligning with a significant difference in beta diversity observed between groups (Bray–Curtis, *P* = 0.004) (**b**). This was not maintained when evaluating specific treatment phases. Alpha diversity for TPN (**c**), EN (**d**) and both groups (**e**) showed significant decreases in early- and mid-phases in both groups (TPN: *P* = 0.029 and *P* = *0.050*; EN: *P* < 0.0001). No significant differences were observed between groups within each treatment phase

Next, the gut microbiome composition was compared between evaluable participants in this trial to explore microbial differences in TPN vs. EN participants (Fig. 3a). Thirty-seven OTUs were differentially abundant between TPN and EN groups (Table S3), with the EN group identified to have a microbiome significantly enriched for 22 OTUs, including *Parabacteroides distasonis* (368.6-fold increase, *P* < 0.0001) and *Staphylococcus haemolyticus* (247.24-fold increase, *P* = 2.13E^−33^). The gut microbiota of people in the EN group was deficient in 15 OTUs, including *Lactobacillus crispatus* (75.6-fold decrease, *P* < 0.0001) and *Parabacteroides johnsonii CL02T12C29* (57-fold decrease, *P* < 0.0001). Accordingly, PERMAOVA (Bray–Curtis) identified significant clustering in microbiome structure based on nutrition (TPN vs. EN, *P* = 0.004); however, this was not maintained with D_0.5_UniFrac, nor was it maintained when comparing treatment groups within auto-HCT treatment phases.

#### Factors explaining gut microbiota variation and dynamics

**Fig. 4:**
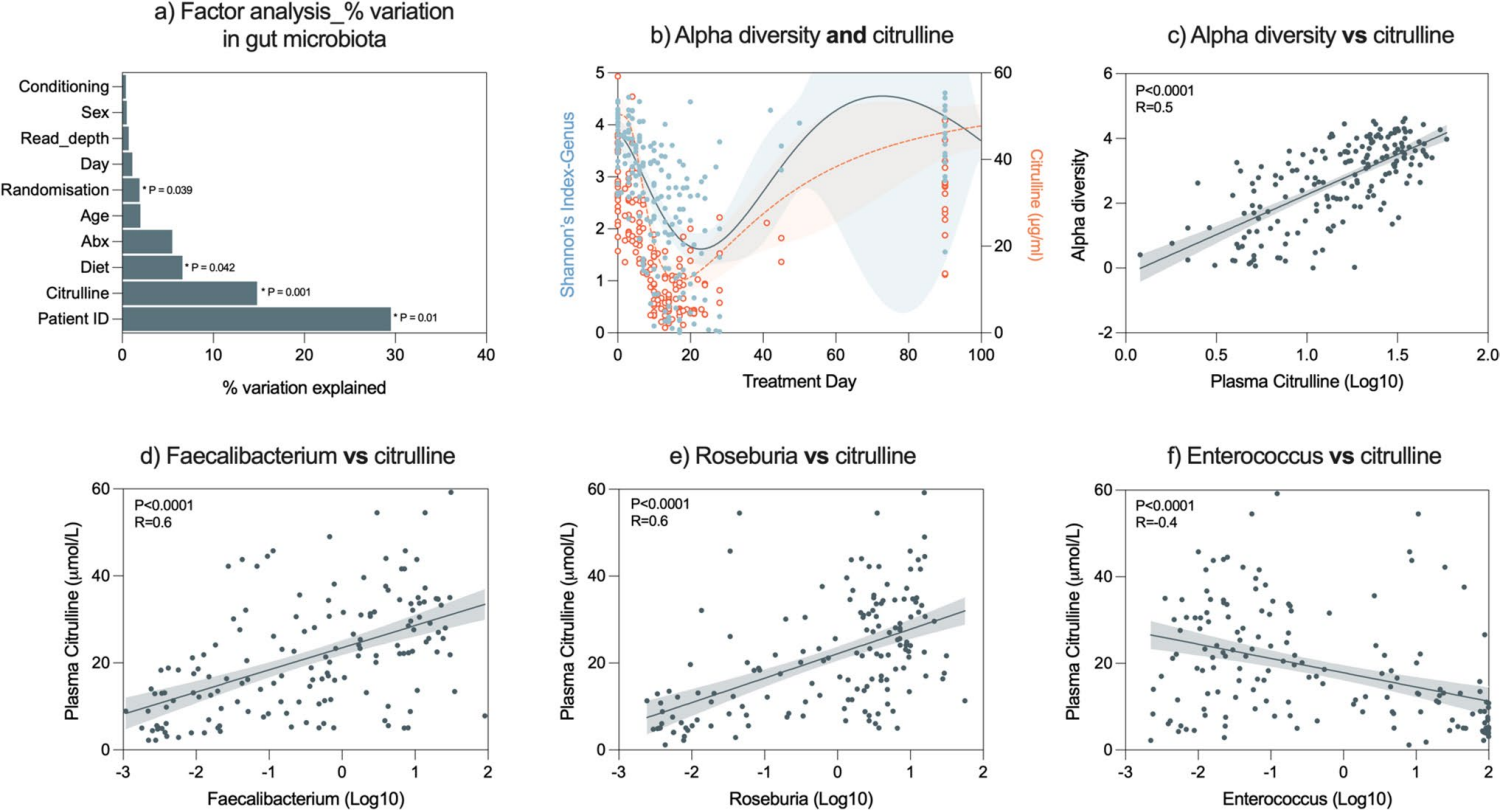
Relationship between faecal microbiome and intestinal mucosa. PERMANOVA analysis describing the percentage of variation (in beta diversity) attributed to various microbial insults, identified plasma citrulline and diet as the most significant sources of variation (**a**, *P* = 0.001, *P* = 0.042, respectively). The most influential factor, plasma citrulline, significantly correlated with alpha diversity (**b**, **c**). Simple linear regression also identified significant correlations between plasma citrulline and *Fecalibacterium* (**d**, *P* < 0.0001), *Rosebria* (**e**, *P* < 0.0001) and *Enterococcus* (**f**, *P* < 0.0001). All correlation coefficients calculated for non-parametric data (Spearman’s coefficient). Simple linear regressions shown with 95% CI on log10 scale

Recognising the breadth and resolution of clinical and biological metadata available in this study, we next aimed to explore the proportional impact of various parameters on the composition of the faecal microbiome using PERMANOVA. The gut microbiome is highly individual specific, and so unsurprisingly, the patient itself (i.e. collectively influence of all factors) explained the majority of the variation (Fig. 4a, P = 0.01). However, when evaluating individual factors, plasma citrulline was the most dominant factor, identified to explain 14.8% of the variation in the composition of the faecal microbiome (*P* = 0.001). Plasma citrulline is a clinically utilised biomarker of GI-M, validated specifically in the context of auto-HCT for its specificity for objectively defining the extent and duration of mucosal injury [22]. It is an amino acid exclusively produced by enterocytes; hence, when enterocyte mass is decreased (in the case of GI-M), plasma citrulline levels are also detectably decreased. For this reason, citrulline is able to determine GI-M without interference, unlike other biomarkers of mucosal injury/inflammation such as Il-6/TNF. Diet (categorised as EN, TPN, normal oral or nil on *the day of the sample collection*) and the randomisation group (EN vs. TPN, *independent* of what diet the participant was consuming when the sample was collected) were also identified to explain variation in the faecal microbiome (6.6% and 1.9%, respectively). Accordingly, plasma citrulline and alpha diversity dynamics were significantly correlated (Fig. 4b, c, R = 0.5, *P* < 0.0001). Of all microbial taxa, the most significant correlations with citrulline were identified for *Fecalibacterium* (Fig. 4d, R = 0.6,* P* < 0.0001), *Roseburia* (Fig. 4e, R = 0.6, *P* < 0.0001) and *Enterococcus* (Fig. 4f, R =  − 0.4, *P* < 0.0001).

The relationship between citrulline and the microbiome highlights the complex interplay between the mucosa and microbes [23]. We therefore attempted to dissect the temporal dynamics of the faecal microbiome and plasma citrulline relative to one another. Cross-correlation analysis between the time course of citrulline and alpha diversity again confirmed that the two parameters were highly correlated (Figure S2). Analysing the correlation between the entire patient population’s citrulline and alpha diversity reveals that citrulline’s rate of change was observed first, with a maximum correlation at Lag − 9 (Figure S2). This suggests that changes in citrulline precede alterations in alpha diversity by 9 days.

Next, we investigated the relationship between citrulline and alpha diversity in individual patients. Cross-correlation analysis was conducted individually on a subset of *N* = 28 patients with *N* ≥ 3 data points (Figure S3). A positive and significant correlation was identified for the patients, indicating a consistent relationship between these variables across the study cohort. Application of three distinct machine learning models (regression, transformer and deep neural network (DNN)) demonstrated that each was able to predict microbial diversity based on citrulline levels with comparable *R*^2^ values (Table S4/5). However, the deep neural network (DNN) model had the lowest error values, suggesting superior performance.

## Discussion

Malnutrition is a common and difficult-to-manage complication of auto-HCT, especially when patients have severe gastrointestinal mucositis (GI-M). Since Weisdorf et al*.* reported an increase in overall survival in auto-HCT [24] recipients receiving TPN, this intervention has remained the preferred method of nutritional support despite its potential risks, including fluid overload, gut atrophy and hepatotoxicity [25–30]. This trial aimed to identify the optimal nutritional support in adult patients receiving an auto-HCT. Our results underscore the challenges in providing EN in adults, with EN not tolerated in the majority of patients. However, whether EN (when delivered in a more acceptable manner) is inferior to TPN cannot be confirmed. Despite this, this trial provided a rich environment to evaluate microbial dynamics in ACHT recipients. Consequently, we were able to show that GI-M is a major driver of validating recent preclinical findings [31] and reinforcing the importance of maintaining mucosal integrity after chemotherapy.

The practical challenges in providing EN in adults were surprising given its success in pediatric patients. In fact, EN has emerged as the preferred nutritional support in children with GI-M based on a small number of studies that confirmed its efficacy in limiting nutritional decline, whilst proving feasible and well tolerated during auto-HCT [16, 17, 32–34]*.* However, it is also important to acknowledge the RCT by Andersen and colleagues [7] which, similarly to our study, reported on the complexities of administering EN in allo-HCT recipients. Certainly, the allo-HCT setting is more complex than the auto-HCT, and Andersen and colleagues adopted strict randomisation criteria which excluded people with GI-M. Hence, only *N* = 9 participants were randomised and of the *N* = 5 randomised to receive EN, all transitioned to parenteral nutrition due to gastrointestinal intolerance. Although Andersen and others [8] have highlighted the challenges of EN in adults, we designed our study to overcome these challenges by inserting the NGT and starting EN at the end of the conditioning regimen just before cytopenia. Despite this, recruitment was difficult and lengthy (2014–2018), with patients reluctant to participate due to the fear of NGT insertion in anticipation of a physically and emotionally high-impact chemotherapy course. Patients clearly had a preference for intravenous nutrition, a preference that may be related to inherent biases/discomfort surrounding EN within the patient’s caregiver team [8, 35]. Moving forward, it may be relevant to consider further modifications to EN delivery (e.g. overnight infusion, altered duration or changes in the type of NGT used) to optimise tolerability. To appropriately identify these refinements, significant engagement with patients, healthcare providers and caregivers, and genuine co-design efforts would be ideal to ensure the patient perspective is adequately integrated into decision-making.

Whilst our data largely reflect the poor tolerance of EN, they also suggest a lack of benefit of EN with regard to weight loss and MUAC. Of course, these conclusions must be made in light of the insufficient power of our study and that most patients got EN only for a limited period, instead of during the whole study period. These results are disappointing in the sense that EN might have several advantages based on data from animal models (e.g. preservation of gut mucosa, adequate amino acid and glucose absorption in G-IM) and clinical studies (e.g. improvement of haematopoietic and gut microbiome recovery) [10–14, 36, 37]. In line with these studies, we did observe differences in gut microbiome community structure in EN vs. TPN participants; however, whether these were ‘beneficial’ or ‘detrimental’ changes is unclear, as there was no difference in alpha diversity and no consistency in the microbes that were differentially abundant in EN vs. TPN participants. For example, differential abundance analysis showed that the gut microbiome of TPN participants was enriched for *Enterococcus faecalis*, a known pathogen implicated in BSI etiology [38, 39]. This supports previous data that show TPN deprives the gut microbiome of critical host-derived fuels (i.e. dietary fibre), resulting in a loss of colonisation resistance [40]. However, equally, the EN group had a microbiome enriched for *Enterococcus faecium*, a microbe already identified to dominate the faecal microbiome in immunocompromised allo-HCT recipients [41].

Understanding the clinical implications of these microbial changes is challenging, particularly when attempting to align outcomes with individual microbial taxa. However, it is of interest to note that the *Parabacteroidetes* genus (*Parabacteroidetes disatonis*) was enriched in the EN group, aligning with its dependence on fructans [42] which are abundant in the enteral feed. This bacterial taxon has been associated with an increased risk of GvHD in allo-HSCT recipients [43]. Preclinically, this specific bacterium has been implicated in modulating intestinal bile acid metabolism [44] and has, in fact, emerged as a probiotic candidate in other clinical indications (e.g. rheumatoid arthritis) [45]. This underscores the complexity of deciphering beneficial vs. detrimental changes in the gut microbiome related to nutrition, especially with the confounding effects of antibiotics in this patient cohort. However, it is important to note that antibiotics were highly protocolised in this study, with empirical use restricted to ceftazidime—a third-generation antibiotic that is less active than first- and second-generation cephalosporins. Although this is thought to have low activity against anaerobes, data do indicate some activity against commensal in vitro [46]. Nonetheless, in our study cohort, the effects of antibiotics on the microbiome were deemed minimal, but this conclusion must be recognised in light of local antibiotic stewardship protocols in our institute.

In line with our assumption that the antibiotic protocol used in this study had minimal effects on the gut microbiome, PERMANOVA showed that antibiotic use did not have a significant effect on the variation in microbial structure in our study cohort. In contrast, we did demonstrate a significant effect of diet, explaining 6.6% of the variation, aligning with recent reports which position diet as a critical factor that dictates microbial resilience and recovery after chemotherapy [47]. Importantly, dietary intake on the day of faecal sample collection (not their randomisation group) was more strongly aligned with gut microbiome composition/variation compared to randomisation group, emphasising the dynamic nature of the gut microbiome and potential benefits of EN when actually delivered. EN is well-positioned to positively influence microbial and mucosal dynamics, providing critical fuel for microbes, promoting SCFA production and preserving mucus production, all of which are heavily impaired in the setting of TPN. The challenge now is to determine how the benefits of EN can be realised in a clinically-feasible manner. This may simply be a matter of providing EN to patients that are willing and therefore likely to tolerate NGT, and optimising the timing of nutritional support. Given our observations that citrulline remained low at days + 45 and + 90 in some patients, indicating malabsorption that persisted months after transplantation, it is also likely that nutritional support (of some kind, e.g. multidisciplinary nutritional care pathway [48, 49]) be provided well after discharge. However, it is relevant to highlight that the intestinal mucosa is capable of absorbing some nutrients even when GI-M is present [10, 11], and therefore understanding how to optimise nutrition in light of persistent GI-M is key.

Despite diet being a major contributor to microbial composition and dynamics [50], the most influential factor shaping the faecal microbiota composition in our study was the intestinal mucosa, as indicated by the biomarker plasma citrulline. This aligns with recent data published by Anderson and colleagues [24], which identified in a preclinical model that epithelial apoptosis (i.e. GI-M) drives dysbiosis, in particular, the expansion of Enterobacteriaceae. Anderson et al. elegantly showed that the abundance of Enterobacteriaceae dictated the recovery of the intestinal mucosa, reiterating the strong negative correlation we identified between citrulline and *Enterococcus*. Similarly, using apoptosis-deficient mice (*Vil-CreTg/wt::Caspase 3/7 fl/fl*), Anderson et al. showed that dysbiosis and host disease markers were reduced. Building from this, they demonstrated that selectively targeting specific microbes using an antibiotic cocktail (containing the gram-positive targeting antibiotic vancomycin, the gram-negative targeting aztreonam and polymyxin B). When considering these data in light of ours, they strongly position GI-M as the catalyst for detrimental gut microbiome changes and the associated clinical consequences. However, it is important to acknowledge the nuanced differences in findings, with Enterobacteriaceae the focus of Anderson’s work, whilst our data mainly highlight the interaction between the mucosa and *Enterococcus*. Whilst both are taxa with pathogenic potential, they belong to different families; thus, their interaction with the mucosa may differ.

In the context of auto-HCT, the concept that GI-M drives gut microbiome disruption highlights the opportunity to predict or prevent associated events (e.g. BSI) by monitoring or controlling GI-M, respectively. Indeed, we demonstrated using machine learning models that key microbial indices can be predicted based on citrulline dynamics alone. The 9-day lag between mucosal destruction and microbial disruption is clinically interesting as it may enable events associated with gut microbiome damage, such as neutropenic fever, BS, relapse and even GvHD (in allo-HCT), to be predicted without the need for complex microbial sequencing. Monitoring citrulline in auto-HCT recipients is clinically-feasible and routinely performed in our centre, and these data certainly suggest that this strategy could help to identify patients at risk of future microbiome-associated events. Indeed, citrulline has been shown to predict BSI with greater accuracy compared to neutrophil counts in auto-HCT recipients [51], underscoring its clinical utility. Mechanistically, these data also highlight the immense control the mucosa has on gut microbiome dynamics, with plasma citrulline not only reflecting pathophysiological events occurring in real time but also the future implications of these changes. When considered in light of the data from Anderson and colleagues, it is clear that there is strong bi-directional communication between the gut mucosa and the microbiome, with GI-M the likely catalyst of detrimental changes and these microbial events then determining the rate of mucosal recovery. Unfortunately, our study lacked the resolution needed to explore the predictive capacity of the gut microbiome on mucosal recovery (due to sparsity in sampling in late treatment phase). However, it is of interest that when the gut microbiome is intentionally damaged using antibiotics prior to and following chemotherapy, mucosal recovery is heavily impaired; as a result, mortality is increased [52]. This therefore raises the question of how best to support the gastrointestinal microenvironment in the context of auto-HCT and whether targeting the mucosa or microbiome is the most effective. Our findings highlight the importance of gut mucosal stability for microbial health, suggesting that maintaining mucosal integrity may enhance microbial resilience, as demonstrated with anakinra [53]. Importantly, unlike the strategy adopted by Anderson and colleagues (in which mucosal recovery was accelerated by controlling pathogen blooms with antibiotics), anakinra provided equally effective microbial support whilst avoiding the negative consequences of antibiotics (e.g. resistance).

Whilst we have provided both clinical and exploratory insights regarding the use of EN in auto-HCT and related microbial dynamics, these must be interpreted in light of some limitations. Firstly, although we adopted rigorous trial methodology (i.e. an appropriately powered RCT), the small study cohort that occurred as a result of poor EN tolerance resulted in the study ultimately being underpowered to determine a difference in the ∆body weight and ΔMUAC. Hence, our conclusions regarding EN efficacy (or lack thereof) should be taken with caution. Similarly, whilst citrulline is considered the most sensitive and accurate biomarker of GI-M [22], other biomarkers (e.g. CRP, albumin) may have shed more light on the effects of nutritional intervention. However, given the poor tolerability of EN, the added value of these analyses is likely limited. With respect to our exploratory microbiome findings, again, the small study cohort undermined our ability to draw robust conclusions regarding microbiota differences between EN and TPN groups. Nonetheless, the entire study cohort and longitudinal sampling provided exceptional resolution when evaluating microbial dynamics, enabling interrogation of the most dominant factors shaping its composition. Whilst we provide important insights on the *sequence* of mucosal and microbial disruption, which add validity to the findings of Anderson and colleagues, it must be made clear that these findings do not infer causality. However, it is important to consider the complexities of determining causality with respect to the microbiome, as well as the challenges of our clinical scenario. Auto-HCT is an intense procedure, and patients are extremely vulnerable for several weeks, at risk of BSI/sepsis and other lethal complications. It is therefore impossible to restrict all confounding factors, such as antibiotics, or perform highly invasive assessments that may be needed to move closer to causality. This underscores the relevance and timeliness of the findings by Anderson, which, when combined with our clinical data, provide a strong case for GI-M being the catalyst of gut microbiome changes in auto-HCT recipients. Finally, the use of machine learning models, in particular more complex models such as transformers, is novel but has some practical limitations specifically for smaller datasets or ‘simpler’ relationships. Whilst the transformer did not significantly outperform the simpler models used (e.g. linear regression, DNN), its inclusion allowed us to evaluate its feasibility for this type of data. Future studies may consider including a more comprehensive battery of models (e.g. random forests, gradient boosting, sequential models) to identify the best performing, but ultimately most feasible model.

## Supplementary Information

Below is the link to the electronic supplementary material.

ESM 1 (DOCX 30.6 KB)

ESM 2 (DOCX 33.4 KB)

ESM 3 (DOCX 1.42 MB)

## Data Availability

No datasets were generated or analysed during the current study.
