# Supplementary material for: Enteral versus parenteral nutrition in auto-HCT: a randomized controlled trial on clinical outcomes and gut microbiome dynamics
Source: Support Care Cancer. 2025 Sep 19;33(10):865. doi: 10.1007/s00520-025-09882-z (PMC12449397; doi:10.1007/s00520-025-09882-z)
Supplement: Supplementary file 1 — (DOCX 30.6 KB) [file 520_2025_9882_MOESM1_ESM.docx]

**Supplementary methods**

*Provision of clinical nutrition*

The daily individual dietary need was calculated by the dietician. Energy requirements were calculated using the WHO formula ’85 TEE with an additional factor 30%. Protein requirements were calculated as 1.2 gram/kg with a correction for underweight (BMI < 20 kg/m^2^) and overweight (BMI > 27.5 kg/m^2^). For all patients, feeding (EN or TPN) was started at day 4 (after nausea and vomiting related to the chemotherapy and before low platelet levels and mucositis) after starting chemotherapy and was still started if patients had a reasonable oral intake. For patients randomized to TPN, a standard solution prepared by the department of pharmacy of the Radboudumc was infused continuously via CVC with standard additives of trace elements (Addamel®, Fresenius Kabi) and vitamins (Cernevit™ Baxter) until the CVC was removed as standard practice, at time of engraftment. For patients randomized to EN, Survimed® (Fresenius Kabi) - an elementary tube feed - was administered via a NGT. The product characteristics of Survimed® are enclosed (Table S1).

In case of CVC loss (by any cause), TPN was stopped earlier. In case of intolerance of enteral feeding, continuing EN at the highest tolerated rate was aspired. In case of NGT loss within 48 hours after placement or when EN was not tolerated during the first five days, the patient was switched to TPN and was considered a failure of EN. Intolerance of EN was defined as either exacerbation of gastrointestinal symptoms unable to be controlled by medication (anti-emetics), intolerance to the nasogastric tube or NGT loss. Please see Supplementary Methods for an overview of supportive care given, including antibiotic prophylaxis and empiric use.

*Secondary endpoints*

Secondary endpoints were differences seen in treatment-related complications, including severity of IM, oral mucositis (OM), febrile neutropenia, blood stream infection (BSI), length of hospital stay and total calory intake. Nutritional status was recorded at day of admission and evaluated two times a week in the morning by means of bodyweight and MUAC. MUAC was determined by the circumference of the left upper arm, measured at the mid-point between the tip of the shoulder and the tip of the elbow (olecranon process and the acromion). The severity of IM was assessed three times per week by measuring citrulline levels to capture the citrulline mean nadir. Plasma citrulline, a validated biomarker of enterocyte mass and IM severity, was assessed in 30 μL of plasma (isolated from whole blood) using automated UPLC tandem mass-spectrometry (Waters, Milford, USA) as previously described (1)*.* Clinically, oral mucositis (OM) was assessed and graded by dedicated nursing staff in accordance with the Daily Mucositis Score (DMS) (2). Neutropenic fever was defined as a single axillary temperature ≥ 38.5°C and an absolute neutrophil count (ANC) < 0.5 x 10^9^/L. BSI was defined as clinical symptoms (i.e. fever) in the presence of bacteremia. Bacteremia was defined as a common commensal organism (e.g. coagulase-negative Staphylococci [CoNs]), isolated from a blood culture on two occasions, or a recognized pathogen isolated from one blood culture. The day of engraftment for granulocytes was defined as the first day on which neutrophil count was greater than ≥0.5 x 10^9^/L. Length of hospital stay was recorded from the day of admission. The total calorie intake from start of nutrition to day +28 was calculated for both groups. After hospital discharge, patients were monitored in the outpatient clinic on post-AHCT day +45 and +90 after starting chemotherapy for measuring bodyweight, MUAC and laboratory tests (citrulline, C-reactive protein (CRP)).

*16S rRNA sequencing and bioinformatics*

DNA was extracted from fecal samples using the double bead-bester procedure adapted from Yu and Morrison (2004) and QIAamp DNA Stool Minikit guidelines (Qiagen, Hilden, Germany)[15](See supplementary methods). Polymerase chain reaction (PCR) was used to amplify V3 and V4 of the 16S rRNA gene, with DNA samples diluted to 100 ng/μl for all PCR amplification procedures. Size selection and fragment removal was achieved using AMPure XP beads (Beckman Coulter, Brea California, USA). DNA was diluted to a working concentration of 2 mM and 5 μl of samples were pooled to form a single library and stored at -20 C until sequencing. Sequencing was performed using the MiSeq Benchtop Next Generation Sequencer (Illumina) following manufacturer’s guidelines. Raw data were analyzed using the Qiagen CLC Software Package V21.0.03. Raw Illumina files were uploaded into the Qiagen CLC Software Package and trimmed using quality scores (limit set to 0.05) and ambiguous nucleotides set to a maximum of 2. Short reads (<5) were discarded and samples with sufficient coverage were then assigned to appropriate operational taxonomic units (OTUs) using the SILVA rRNA reference library (version 132, 99% coverage) in the Qiagen CLC Software (default clustering pipeline).

Alpha and beta diversity (from 16S rRNA sequencing data) were calculated using the default pipelines in the Qiagen CLC Software Package based on phylogenic diversity and UniFrac distances. The phylogenic tree was reconstructed using a Maximum Likelihood approach based on Multiple Sequence Alignment and the OTU sequences generated by MUSCLE in the workbench. For alpha diversity, the rarefaction analysis maximum depth was set to 5000. Alpha diversity was compared across groups using a one-way ANOVA. PERMANOVA analysis was used to measure effect size and significance of beta diversity between groups. The *envfit* function in the vegan R library (vegan v2.6.2, R v4.2.0) was used to identify the percent of variation explained by different host factors in the data. Correlations analyses were calculated in MATLAB 32021, 64-bit. The correlation between citrulline levels and alpha diversity was analyzed using the Pearson correlation coefficient. A two-tailed test was performed to assess the significance of the correlation coefficient. In addition to the correlation analysis, three machine learning (ML) models were developed to predict alpha diversity based on citrulline levels. These models were applied to participant data where ≥3 repeated samples with data were collected. The ML models utilized in this study were *i*) regression, *ii*) transformers, and *iii*) deep neural networks. These models were selected due to their ability to capture complex relationships and patterns in the data. More detailed methods can be found in Supplementary Methods.

*Machine learning models*

In addition to the correlation analysis, three machine learning (ML) models were developed to predict alpha diversity based on citrulline levels. These models were applied to participant data where ≥3 repeated samples with data were collected. The ML models utilized in this study were *i*) regression, *ii*) transformers, and *iii*) deep neural networks. These models were selected due to their ability to capture complex relationships and patterns in the data.

Regression Model*:* A regression model was constructed to predict alpha diversity as a continuous variable based on citrulline levels. The model was trained utilizing the continuous citrulline data as input features and the corresponding alpha diversity measures as the target variable. The Linear regression model was created using the ‘LinearRegression’ class from the ‘sklearn.linear_model’ module. The model was fitted to the training data using the ‘fit’ method, which enables the model to learn the underlying relationships between citrulline levels and alpha diversity. By analyzing the training data, the model determined the optimal weights and bias values for the linear equation that best predicts alpha diversity based on citrulline levels.

Transformers: Transformers(4), a type of deep learning architecture known for their effectiveness in natural language processing tasks, were adapted for the prediction of alpha diversity. The transformer model employed a self-attention mechanism to capture relationships between citrulline levels and alpha diversity. The model was trained using the citrulline levels as input and the corresponding alpha diversity measures as the target.

In addition to transformers proficiency in natural language processing, the transformer architecture's attention mechanisms and deep contextual understanding make it applicable to time series forecasting as well. By representing time series data as a sequence of tokens, such as historical observations or features, our transformer model can encode the temporal dependencies and relationships within the citrulline and alpha diversity data. This allows the model to capture long-term dependencies and effectively learn patterns and trends in the time series. The transformer-based architecture, with its multiple encoder layers, attention mechanisms, and residual connections, enhances the model's ability to extract meaningful temporal information and make accurate predictions for future time points.

Deep Neural Networks (DNNs)*:* DNNs, a type of artificial neural network with multiple hidden layers, were employed to predict alpha diversity capable of learning complex patterns and relationships in the data. The architecture of the DNN model consisted of multiple layers with varying numbers of neurons. The model was trained using the citrulline levels as input features and alpha diversity as the target variable.

The DNN model was constructed using the Keras framework. The model architecture consisted of three layers: an input layer with 32 neurons and rectified linear unit (ReLU) activation function, a hidden layer with 16 neurons and ReLU activation function, and an output layer with a single neuron and linear activation function. To train the DNN model, the model was compiled with the ADAMoptimizer and the mean squared error (MSE) loss function. The model was then trained on the input data (X) and target data (y) for a specified number of epochs and a batch size of 32.

Statistical analyses and ML model developments were conducted using Python programming language and libraries, such as scikit-learn, TensorFlow, seaborn, keras, transformers, numpy, pandas, scipy, and matplotlib). The dataset was randomly split into training and testing sets to train and evaluate the ML models. The training set (80%) was used to optimize the model parameters, while the testing set (20%) provided an independent dataset for assessing the model's generalization performance. The models' performance was evaluated using appropriate evaluation metrics specific to each ML model, as described above. To assess the performance of each ML model, we utilized evaluation metrics such as Root mean squared error (RMSE), MSE, mean absolute error (MAE), and R-squared ($R^{2}$). Detailed explanations and formulas for these metrics can be found in Table S5.

| **Table S5: Evaluation metrics** | |
| --- | --- |
| Root mean squared error (RMSE) | RMSE = $\sqrt{\frac{1}{n}\sum_{i=1}^{n} {(y_{i}-\hat{y}_{i})}^{2}}$ |
| Mean absolute error (MAE) | MAE = $\frac{1}{n}\sum_{i=1}^{n} (y_{i}-\hat{y}_{i})$ |
| Mean squared error (MSE) | MSE = $\frac{1}{n}\sum_{i=1}^{n} {(y_{i}-\hat{y}_{i})}^{2}$ |
| R-squared value | $R^{2}=1-\frac{Sum of squares of residuals}{Total sum of squares}$ |

Where $n$ is number of data points, $y_{i}$ observed values, and $\hat{y}_{i}$ predicted values.

1. Demacker PN, Beijers AM, van Daal H, Donnelly JP, Blijlevens NM, van den Ouweland JM. Plasma citrulline measurement using UPLC tandem mass-spectrometry to determine small intestinal enterocyte pathology. J Chromatogr B Analyt Technol Biomed Life Sci. 2009;877(4):387-92.

2. Donnelly JP, Muus P, Schattenberg A, De Witte T, Horrevorts A, DePauw BE. A scheme for daily monitoring of oral mucositis in allogeneic BMT recipients. Bone Marrow Transplant. 1992;9(6):409-13.

3. Averbuch D, Orasch C, Cordonnier C, Livermore DM, Mikulska M, Viscoli C, et al. European guidelines for empirical antibacterial therapy for febrile neutropenic patients in the era of growing resistance: summary of the 2011 4th European Conference on Infections in Leukemia. Haematologica. 2013;98(12):1826-35.

4. Vaswani A, Shazeer N, Parmar N, Uszkoreit J, Jones L, Gomez AN, et al. Attention is all you need. Advances in neural information processing systems. 2017;30.
