# Supplementary material for: Enteral versus parenteral nutrition in auto-HCT: a randomized controlled trial on clinical outcomes and gut microbiome dynamics
Source: Support Care Cancer. 2025 Sep 19;33(10):865. doi: 10.1007/s00520-025-09882-z (PMC12449397; doi:10.1007/s00520-025-09882-z)
Supplement: Supplementary file 2 — (DOCX 33.4 KB) [file 520_2025_9882_MOESM2_ESM.docx]

| **Table S1: Composition of Survimed® (Nutrients per 100 ml)** | | |
| --- | --- | --- |
|  | *Unit* | *Amount* |
| **Energy** | Kcal | 100 |
|  | Kj | 420 |
| **Protein** | G | 4.5 |
| **Carbohydrates** | G | 14.3 |
| Sugars | G | 1.1 |
| -fructose | G | 0.0 |
| -lactose | G | <0.1 |
| **Fat** | G | 2.8 |
| saturated | G | 0.15 |
| -MCT | G | 1.44 |
| common unsaturated | G | 0.61 |
| complex unsaturated | G | 0.60 |
| -linoleic acid | G | 0.45 |
| -α-linoleic acid | G | 0.08 |
| -EPA + DHA | G | 0.04 |
| -ω6:ω3 fatty acids |  | 3.5:1 |
| Cholesterol | Mg | 0.5 |
| **Fibres** | G | 0.08 |
| -cellulose | G | 0.08 |
| **H2O** | Ml | 85 |
| **Osmolarity** | mosmol/l | 300 |
| **Osmolarity** | mosmol/kg H2O | 350 |
| **Energy distribution** |  |  |
| Protein%: Fat%: Carbohydrates% |  | 18%:25%:57% |
| **Minerals and Trace elements** |  |  |
| Sodium | Mg | 80 |
| Potassium | Mg | 200 |
| Chloride | Mg | 83 |
| Calcium | Mg | 65 |
| Magnesium | Mg | 25 |
| Phosphorus | Mg | 48 |
| Iron | Mg | 1.3 |
| Zinc | Mg | 1.2 |
| Copper | µg | 133 |
| Manganese | Mg | 0.27 |
| Iodine | µg | 13.3 |
| Fluorine | Mg | 0.13 |
| Chromium | µg | 6.7 |
| Molybdenum | µg | 10 |
| Selenium | µg | 6.7 |
| **Vitamins and other nutrients** |  |  |
| Vitamin A | µg RE | 70 |
| Β-Caroteen | µg | 133 |
| Vitamin D3 | µg | 1 |
| Vitamin E | mg TE | 1.3 |
| Vitamin K1 | µg | 6.7 |
| Vitamin B1 | Mg | 0.13 |
| Vitamin B2 | Mg | 0.17 |
| Niacine | Mg | 1.6 |
| Vitamin B6 | Mg | 0.16 |
| Vitamin B12 | µg | 0.27 |
| Panthothenic acid | Mg | 0.47 |
| Biotine | µg | 5 |
| Folic acid | µg | 26.7 |
| Vitamin C | Mg | 8 |
| Choline | Mg | 36.7 |

| **Table S2: Differentially abundance taxa relative to baseline** | | | |
| --- | --- | --- | --- |
| **OTU name** | **Phase** | **Fold change** | ***adjp Value**** |
| *Uncultured Staphylococcus sp* | Early | 3025.93 | *0.00000* |
| *Enterococcus casseliflavus* | Early | 2942.66 | *0.00000* |
| *Enterococcus sp.* | Early | 336.76 | *0.00000* |
| *Staphylococcus haemolyticus* | Early | 152.49 | *0.00000* |
| *Granulicatella sp. oral clone ASCG05* | Early | 150.49 | *0.00000* |
| *Enterococcus faecalis* | Early | 89.75 | *0.00000* |
| *Staphylococcus sp.* | Early | 82.22 | *0.00000* |
| *Veillonella sp.* | Early | 56.91 | *0.00000* |
| *Streptococcus pneumoniae* | Early | 56.85 | *0.00000* |
| *Lactobacillus crispatus* | Early | 46.92 | *0.00000* |
| *Bacteroides ovatus* | Early | 45.00 | *0.00000* |
| *Enterococcus durans* | Early | 29.20 | *0.00122* |
| *Enterococcus faecium* | Early | 28.73 | *0.00001* |
| *Lactobacillus fermentum* | Early | 27.71 | *0.00000* |
| *Lactobacillus reuteri* | Early | 23.40 | *0.00000* |
| *Streptococcus gordonii* | Early | 20.17 | *0.00002* |
| *Rothia mucilaginosa* | Early | 20.03 | *0.00001* |
| *Enterococcus faecium* | Early | 14.42 | *0.02656* |
| *Lactococcus lactis subsp. lactis* | Early | 12.81 | *0.00091* |
| *Bacteroides massiliensis* | Early | 11.63 | *0.07622* |
| *Lactobacillus gasseri* | Early | 11.62 | *0.00666* |
| *Actinomyces graevenitzii* | Early | 10.51 | *0.01302* |
| *Escherichia coli* | Early | 8.26 | *0.02286* |
| *TM7 phylum sp. oral clone DR034* | Early | 7.39 | *0.03414* |
| *Bacterium LF-3* | Early | -11.06 | *0.00094* |
| *Uncultured Lactobacillaceae bacterium* | Early | -21.14 | *0.00000* |
| *Ruminococcus sp.* | Early | -94.11 | *0.00000* |
| *Enterococcus faecalis* | Mid | 9268.70 | *0.00000* |
| *Uncultured Staphylococcus sp* | Mid | 5641.69 | *0.00000* |
| *Enterococcus casseliflavus* | Mid | 3841.85 | *0.00000* |
| *Lactobacillus casei* | Mid | 2222.12 | *0.00000* |
| *Enterococcus sp.* | Mid | 691.18 | *0.00000* |
| *Staphylococcus haemolyticus* | Mid | 619.48 | *0.00000* |
| *Lactobacillus sp.* | Mid | 528.20 | *0.00000* |
| *Staphylococcus sp.* | Mid | 289.71 | *0.00000* |
| *Enterococcus durans* | Mid | 249.69 | *0.00000* |
| *Enterococcus faecium* | Mid | 169.09 | *0.00000* |
| *Lactobacillus fermentum* | Mid | 115.23 | *0.00000* |
| *Rothia mucilaginosa* | Mid | 104.47 | *0.00000* |
| *Streptococcus gordonii* | Mid | 100.93 | *0.00000* |
| *Bacteroides massiliensis* | Mid | 95.16 | *0.00000* |
| *Enterococcus sp.* | Mid | 90.54 | *0.00000* |
| *Streptococcus pneumoniae* | Mid | 83.52 | *0.00000* |
| *Parabacteroides distasonis* | Mid | 78.89 | *0.00000* |
| *Streptococcus salivarius subsp. thermophilus* | Mid | 59.65 | *0.00000* |
| *Lactobacillus rhamnosus* | Mid | 53.88 | *0.00000* |
| *Enterococcus canis* | Mid | 49.83 | *0.00000* |
| *Parabacteroides johnsonii* | Mid | 40.08 | *0.00000* |
| *Enterococcus xinjiangensis;* | Mid | 20.53 | *0.00065* |
| *Eubacterium siraeum* | Mid | -7.18 | *0.01382* |
| *Eubacterium ramulus* | Mid | -7.87 | *0.08429* |
| *Faecalibacterium sp. canine oral taxon 147;* | Mid | -10.43 | *0.00134* |
| *Uncultured Clostridium sp.* | Mid | -13.07 | *0.00001* |
| *Bacterium LF-3* | Mid | -13.79 | *0.00001* |
| *Uncultured Lactobacillaceae bacterium* | Mid | -15.51 | *0.00000* |
| *Ruminococcus sp.* | Mid | -18.27 | *0.00000* |
| *Eubacterium eligens* | Mid | -20.16 | *0.00000* |
| *Uncultured Ruminococcus sp.* | Late | 79.27 | *0.00000* |
| *Ruminococcus sp.* | Late | 68.63 | *0.00000* |
| *Lactobacillaceae bacterium* | Late | -26.48 | *0.00869* |

| **Table S3: Differentially abundance taxa EN vs TPN** | | |
| --- | --- | --- |
| **OTU name** | **Fold change (EN vs TPN)** | ***p Value*** |
| *Parabacteroides distasonis* | 368.59 | *2.87E-41* |
| *Staphylococcus haemolyticus* | 247.24 | *2.13E-33* |
| *Staphylococcus sp. Bdr7* | 115.86 | *1.92E-26* |
| *Bacteroides thetaiotaomicron* | 57.08 | *2.58E-21* |
| *Lactobacillus fermentum* | 27.84 | *2.39E-15* |
| *Lactobacillus sp* | 27.37 | *6.49E-14* |
| *Granulicatella sp. oral clone* | 22.62 | *5.75E-14* |
| *Enterococcus faecium* | 21.62 | *3.80E-15* |
| *Lactobacillus casei* | 15.36 | *1.92E-07* |
| *[Eubacterium] eligens ATCC 27750* | 11.09 | *7.21E-09* |
| *Veillonella sp. HPA0037* | 11.01 | *3.50E-08* |
| *Enterococcus casseliflavus* | 8.41 | *3.83E-03* |
| *Negativibacillus massiliensis* | 7.99 | *1.22E-06* |
| *Clostridium orbiscindens 1_3_50AFAA* | 7.9 | *1.02E-06* |
| *Enterococcus durans* | 7.65 | *3.75E-05* |
| *Lactobacillus reuteri* | 6.75 | *6.36E-05* |
| *[Eubacterium] siraeum DSM 15702* | 5.94 | *1.01E-04* |
| *Rothia mucilaginosa* | 5.44 | *0.01* |
| *Enterococcus sp. CGLBL213* | 5.17 | *8.37E-04* |
| *Lactobacillus gasseri* | 5.17 | *0.03* |
| *Turicibacter sp. H121* | 4.54 | *8.10E-03* |
| *Clostridium paraputrificum* | 4.23 | *0.02* |
| *Bacterium NLAE-zl-G24* | -5.08 | *0.01* |
| *Enterococcus casseliflavus* | -5.39 | *8.40E-03* |
| *Finegoldia magna* | -5.83 | *1.69E-03* |
| *Acidaminococcus intestini DSM 21505* | -6.15 | *4.26E-03* |
| *Coprococcus sp. HPP0074* | -6.93 | *3.37E-03* |
| *Bacteroides ovatus V975* | -7.56 | *8.73E-05* |
| *Bacteroides eggerthii DSM 20697* | -8.13 | *7.36E-06* |
| *Bacteroides sp. 3_2_5* | -9.01 | *1.90E-07* |
| *Bacteroides uniformis CL03T12C37* | -10.39 | *4.85E-08* |
| *Bacteroides massiliensis* | -11.3 | *6.91E-05* |
| *Alistipes sp. AL-1* | -15.6 | *1.46E-08* |
| *Enterococcus faecalis* | -19.06 | *7.70E-06* |
| *Bifidobacterium ramosum* | -23.37 | *1.39E-11* |
| *Parabacteroides johnsonii CL02T12C29* | -57.01 | *6.79E-23* |
| *Lactobacillus crispatus* | -75.64 | *8.55E-22* |

| **Table S4: Evaluation Metrics Comparison: RMSE, MAE, MSE, and** $\mathbf{R}^{\mathbf{2}}$**scores for Regression, Transformers, and DNN models.** | | | | |
| --- | --- | --- | --- | --- |
|  | RMSE | MAE | MSE | $R^{2}$ |
| Regression | 1.05 | 0.91 | 1.19 | 0.38 |
| Transformers | 1.37 | 1.13 | 1.88 | 0.41 |
| DNN | 1.04 | 0.83 | 1.08 | 0.39 |

| **Table S5: Evaluation metrics** | |
| --- | --- |
| Root mean squared error (RMSE) | RMSE = $\sqrt{\frac{1}{n}\sum_{i=1}^{n} {(y_{i}-\hat{y}_{i})}^{2}}$ |
| Mean absolute error (MAE) | MAE = $\frac{1}{n}\sum_{i=1}^{n} (y_{i}-\hat{y}_{i})$ |
| Mean squared error (MSE) | MSE = $\frac{1}{n}\sum_{i=1}^{n} {(y_{i}-\hat{y}_{i})}^{2}$ |
| R-squared value | $R^{2}=1-\frac{Sum of squares of residuals}{Total sum of squares}$ |

*Where* $n$ *is number of data points,* $y_{i}$ *observed values, and* $\hat{y}_{i}$ *predicted values.*
