# Supplementary material for: Enteral versus parenteral nutrition in auto-HCT: a randomized controlled trial on clinical outcomes and gut microbiome dynamics
Source: Support Care Cancer. 2025 Sep 19;33(10):865. doi: 10.1007/s00520-025-09882-z (PMC12449397; doi:10.1007/s00520-025-09882-z)
Supplement: Supplementary file 3 — (DOCX 1.42 MB) [file 520_2025_9882_MOESM3_ESM.docx]

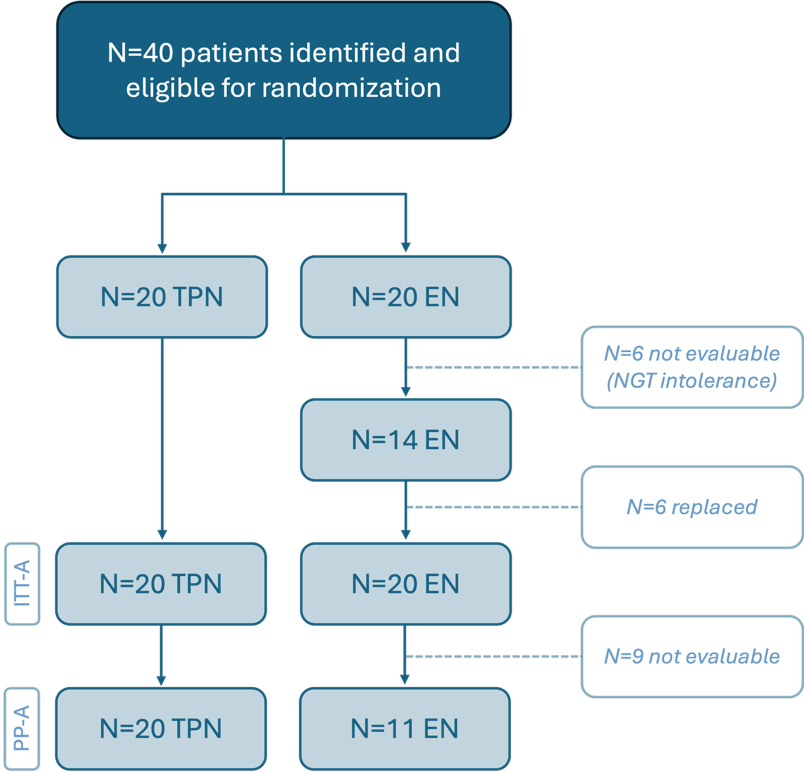


**Figure S1**: Flow chart of number and distribution of patients participating in the study. N=6 patients were replaced because of withdrawal (NGT intolerance within 48 hours) and that N=9 patients were not evaluable (treatment failure because EN given < 5 days because of intolerance or tube loss) and were therefore excluded in the final per protocol analysis. These participants were also excluded from exploratory microbial analyses where comparisons were drawn between TPN and EN groups. *ITT-A = intention to treat analysis; PP-A = per protocol analysis.*


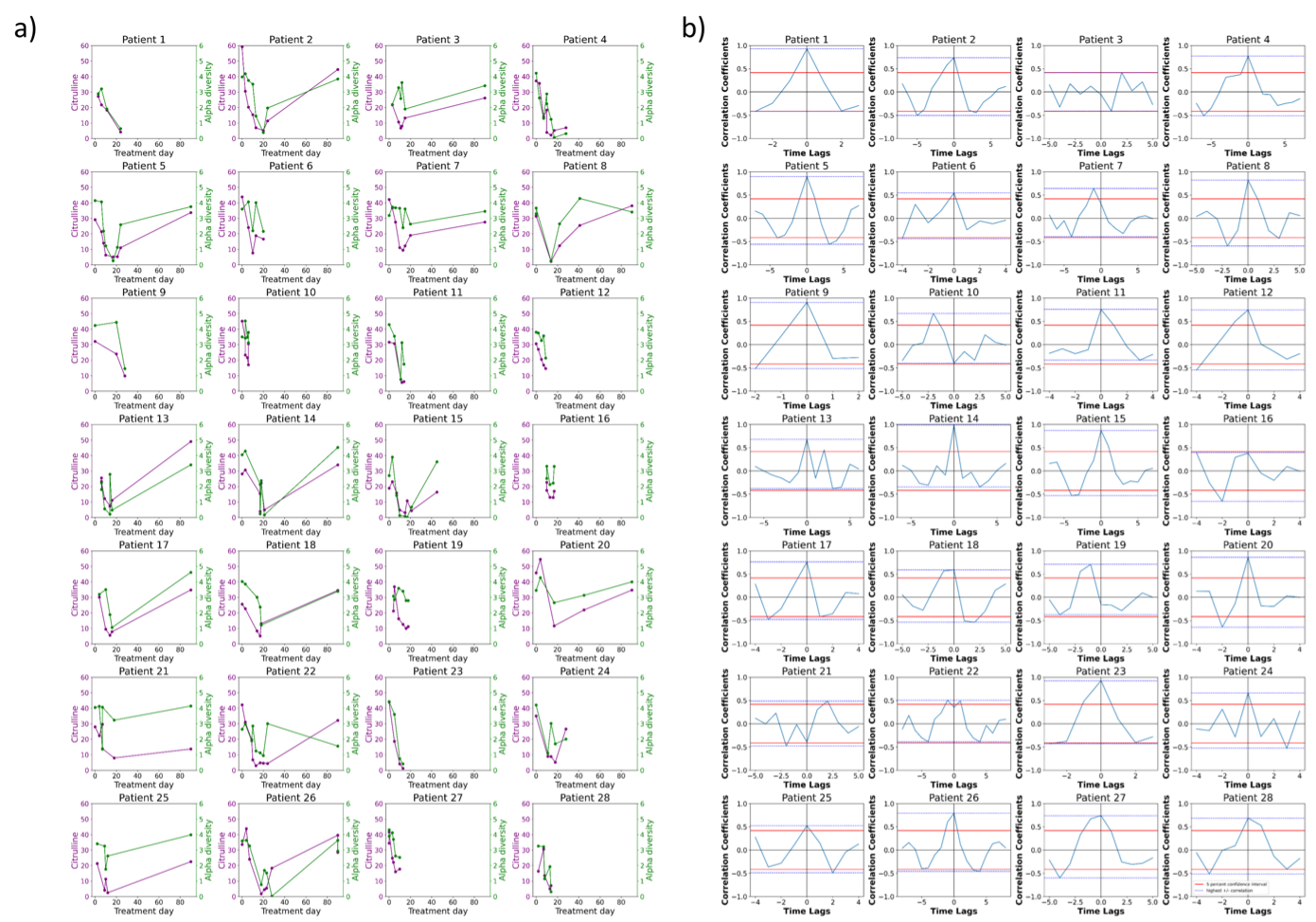


**Figure S2:** Temporal dynamics of mucosal injury and microbial disruption. a) Numerical gradient of alpha diversity and citrulline, showing that first the rate of citrulline changes (crosses zero line), then the rate of alpha diversity follows the change in the same direction (either increasing or decreasing). b) Sample cross correlation between alpha diversity and citrulline illustrates these two timeseries are highly correlated. The dark grey arrow shows the maximum correlation at Lag = -9 indicating the alpha diversity follows the changes in citrulline with a 9 day delay. The upper confidence bound is shown by the horizontal blue line. The orange arrow points day 18 where cross correlation plot is beyond the confidence bound.


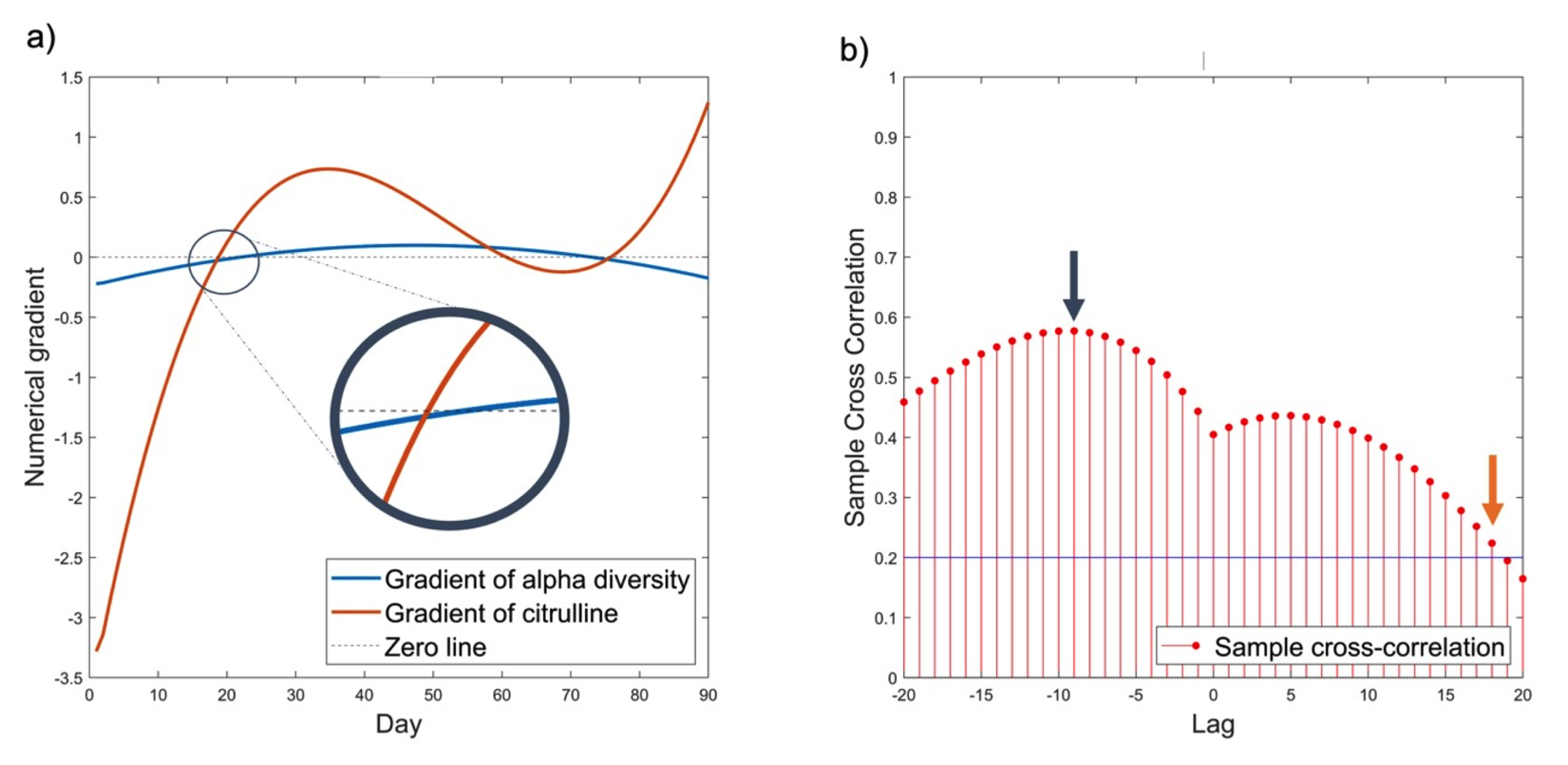


**Figure S3:** Interaction between citrulline and alpha diversity indicates consistent and significant correlation. a) Individual plots of citrulline levels and alpha diversity over treatment days in auto-HCT recipients. Citrulline is shown in purple (left y-axis) and alpha diversity is shown in green (right y-axis). The x-axis represents the treatment days, providing a temporal perspective of the changes in citrulline levels and alpha diversity throughout the treatment period. Each subfigure illustrates the unique correlation pattern between citrulline levels and alpha diversity for a specific patient, offering a comprehensive visual analysis of the dataset. b) Individual cross correlation analysis between citrulline and alpha diversity. The blue dotted lines indicate the highest positive and negative correlation values observed across all patients. The red solid lines represent the 5% confidence interval, highlighting the range of correlations expected by chance alone. For all patients, with the exception of 16, the blue line extends beyond the red line at lag 0, indicating statistically significant positive correlations.
